# Adult aorta coarctation associated with double superior vena cava

**DOI:** 10.11604/pamj.2018.29.17.14137

**Published:** 2018-01-08

**Authors:** Chtioui Mamoun, Fellat Nadia

**Affiliations:** 1 Cardiologue Interventionnel, Departement de Cardiologie Interventionnelle, 1 Centre Medico Chirurgical, Agadir, Maroc; 2 Professeur de Cardiologie, Cardiologue Interventionnelle, Service de Cardiologie a, CHU Ibn Sina, Rabat, Maroc

**Keywords:** Hypertension, coarctation, stent

## Image in medicine

We report the case of a 45 years old man with no individual's history, suffering from hypertension recently discovered, admitted to our department for hypertensive urgencies. The clinical exam found a significant difference in blood pressure between the upper and lower limbs. The radial and ulnar pulses are present but the pulses of both lower limbs are abolished. The resting electrocardiogram recorded a regular and sinus rhythm and repolarization disorders laterally. The chest x-ray showed a normal heart volume and diffuse ribs erosions. The Transthoracic echodoppler displayed a normal left ventricular size and systolic function and a major LV enlargement. The ascending aorta was at normal size with the presence of a normal tricuspid aortic valve. The thoraco-abdominal CT (A, B) showed a coarctation of the proximal portion of the descending thoracic aorta and a double permeable superior vena cava. The patient underwent successful endovascular treatment of the coarctation with stent placement (C, D). The particularity of this observation is the late announcement of the coarctation at adult age and its association with a double superior vena cava.

**Figure 1:**
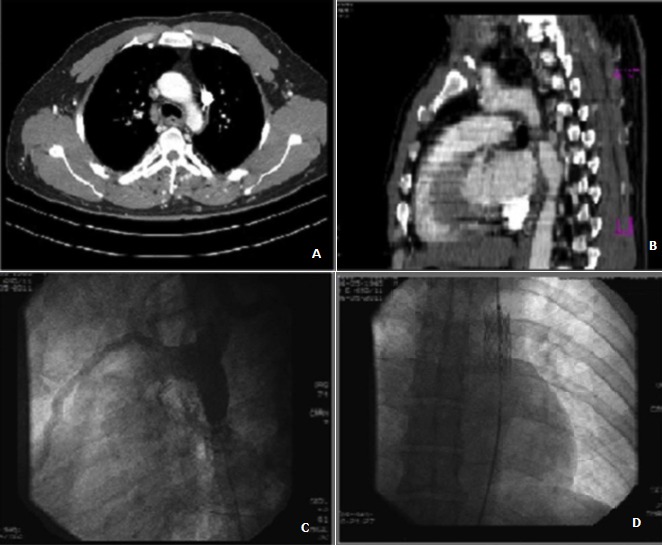
Aorta coartation and stent placement

